# Perceptions of the 2D short animated videos for literacy against chronic diseases among adults with diabetes and/or hypertension: a qualitative study in primary care clinics

**DOI:** 10.1186/s12875-024-02621-z

**Published:** 2024-10-21

**Authors:** Panan Pichayapinyo, Malinee Sompopcharoen, Weena Thiangtham, Jutatip Sillabutra, Phenchan Meekaew, Benyada Bureerat, Armote Somboonkaew

**Affiliations:** 1 Department of Public Health Nursing, Faculty of Public Health, Mahidol University, Bangkok, Thailand; 2 Department of Health Education and Behavioral Sciences, Faculty of Public Health, Mahidol University, Bangkok, Thailand; 3 Department of Biostatistics, Faculty of Public Health, Mahidol University, Bangkok, Thailand; 4 National Electronics and Computer Technology Center, Pathum Thani, Thailand

**Keywords:** Animation, Chronic disease, Health education, Perception, Smartphone

## Abstract

**Background:**

Animation has promise for teaching complex health content through smartphone applications. However, smartphones have had limited use in Thailand for health literacy improvement among adults with chronic diseases. This study aims to explore the perceptions of adults with diabetes and/or hypertension resulting from 2D short animated videos for literacy against chronic disease that are available via smartphones.

**Methods:**

Four animated videos were initially developed based on clinical practice guidelines and nursing experience. Physicians, an expert in health education, and an animation team developed and revised scripts and storyboards of the animated videos. Lastly, videos were validated by physicians and health educators for content breadth and depth and by media experts for motion graphics and illustration. Each video presents a different situation in diabetes, hypertension, missing appointments, and obesity, ranging from 2.18 to 4.14 min in duration. The inclusion criteria were adults 35–70 years old with diabetes or hypertension who received care from primary care units. Thematic analysis was performed on the extracted data.

**Results:**

Twenty participants with a mean age of 58.4 years (SD 6.7; ranged 41–68) were focus-group interviewed. Three animation elements were assessed, including presentation, impacts, and suggestions. Participants had positive responses regarding the presentation (suitability of images and smooth motion graphics, short length of videos, simple language, understandable content, and clear sound) and impacts (recalling information, enhancing engagement, and motivating health awareness and behavior change). Suggested improvements were for a bigger font size for subtitles and considering the use of spoken text instead of on-screen text for recommendations at the end of the animations.

**Conclusions:**

Animated videos are acceptable for delivering health information. Pilot testing animated videos for promoting literacy against chronic diseases in adults with diabetes and hypertension is needed for optimal utility.

## Background

The increasing prevalence of chronic diseases, particularly diabetes and hypertension, is worldwide. According to the World Health Organization report, the mortality rate due to diabetes in lower-middle-income countries (LMICs) increased 13% between 2000 and 2019 [1]. Percentage change in deaths of hypertension-related cardiovascular disease mortality in LMICs increased 29% between 1990 and 2019 [2]. This evidence is consistent with Thai statistics show that the mortality rate of diabetes increased by 17.82%, and hypertension increased by 17.07% between 2018 and 2022 [3]. Promoting behavior changes including healthy diet, physical activity, alcohol reduction, and quit smoking has been recommended to prevent and delay the onset of the diseases [4]. 

Health literacy enhancement for complex behavior changes is critical to medical treatment adherence and self-care, improving health outcomes, delaying adverse outcomes, and decreasing premature death [5–7]. Most programs focused on understanding diseases, complying with medical treatment, and appropriate self-care in eating habits and physical activity. They were conducted in small group educational classes via face-to-face learning [8, 9]. However, these strategies might benefit patients receiving care in hospitals where health professionals can provide periodic educational classes. Specifically, conveying an understanding message to motivate adults with chronic diseases to comply with treatment and sustain self-care is the greatest challenge in Thai primary care clinics, which often have a shortage of health personnel. In 2021, Thai statistics reported one physician per 1680 population and one nurse per 353 population, and this ratio is even lower in primary care clinics [10]. 

Animation is a promising strategy for dealing with complex content, which is challenging to teach and learn worldwide [11]. Animation is described as a type of media with a series of pictorial displays and sounds that make an attractive appearance to learners [12, 13]. According to the Cognitive Theory of Multimedia Learning, people learn information through words and pictures [14]. They learn more when words and pictures are integrated; the words can be verbal or text, and pictures can be any form of illustration, video, or animation. The fundamental concept of the theory is that the learners will attempt to create meaningful connections between words and pictures. In healthcare, evidence is clear regarding changing health behaviors, improving knowledge and health literacy, and adhering to medical prescriptions with moving images and sound compared to traditional education [15–19]. 

Currently, smartphones are tools with which people can easily engage in health education and are used as a channel to deliver audio-visual media for learning [20–22]. Although most animation was developed to convey messages through the internet, there is a limitation to developing animations specifically accessed by smartphones. Therefore, 2D short animated videos that can be accessed via smartphones to convey health messages targeting adults with diabetes and hypertension who receive care at the primary care units were developed by the researchers. Still, there is a need for more knowledge regarding the design and the feasibility of 2D short animated videos before going through further study in Thai communities. This study aims to explore the perceptions of adults with diabetes and hypertension on four animated videos after accessing them via smartphones.

## Methods

### Study design

This exploratory study incorporated a qualitative approach with focus-group interviews and was conducted in October 2023 at two primary care clinics in suburban areas of two provinces near Bangkok, the capital city of Thailand.

### Animation development

**Fig. 1:**
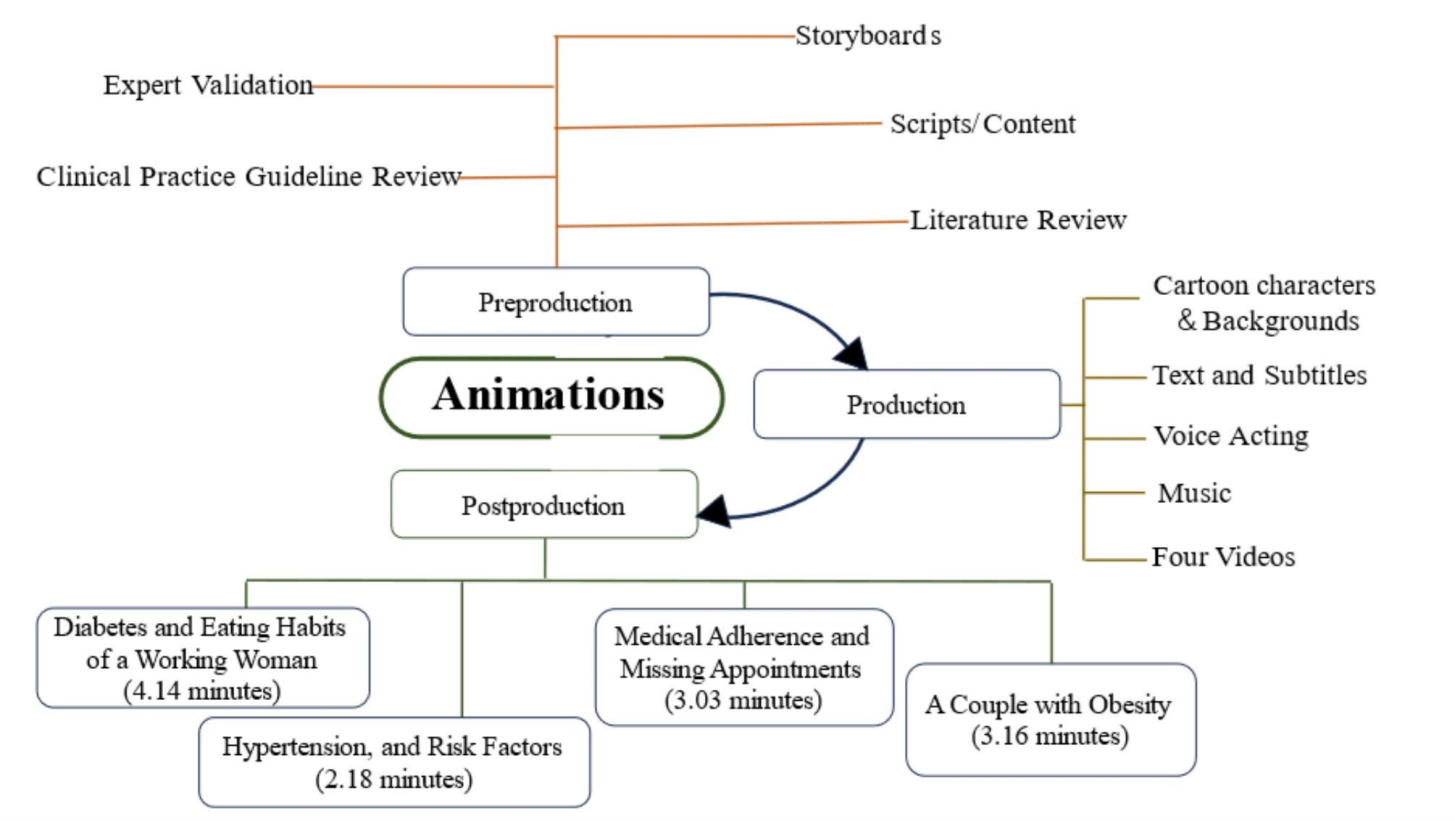
The scope of the study design

The researchers developed animated videos for Literacy Against Chronic Diseases (ALAC) based on the Cognitive Theory of Multimedia Learning as a conceptual framework for watching via smartphones, in which people can easily engage with our animations anywhere and anytime. The process of animated videos development was divided into 3 phases. The scope of the study design is shown in Fig. 1.

In the pre-production phase, four scripts were initially drafted based on the literature review and everyday situations often found in primary care clinics. Working with physicians who are experts in chronic diseases such as diabetes and hypertension and an expert in health education, scripts and storyboards have been revised for content accuracy and conciseness.

**Fig. 2:**
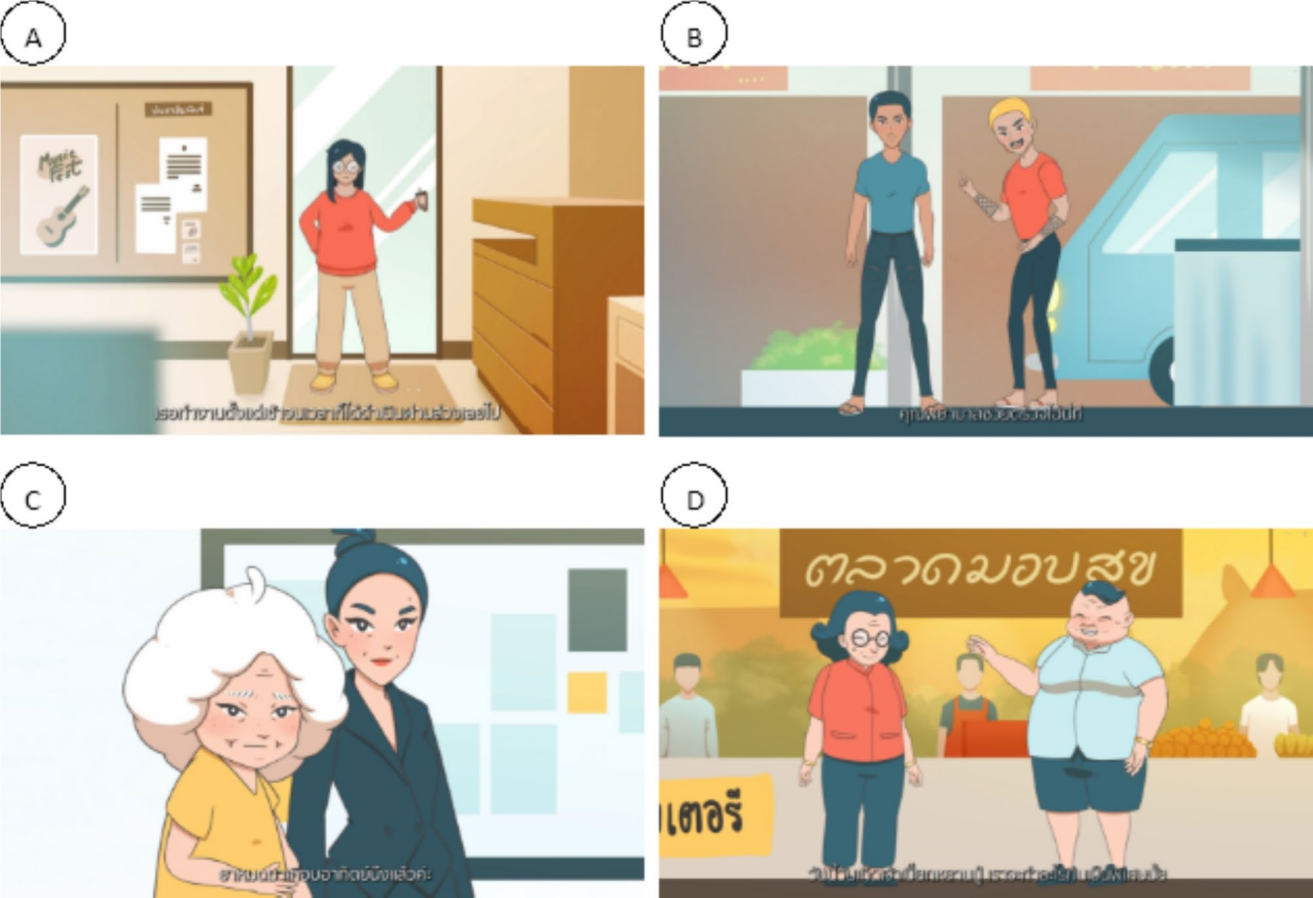
Scenes from (**A**) Video 1: Diabetes and Eating Habits of a Working Woman, (**B**) Video 2: Hypertension, and Risk Factors, (**C**) Video 3: Medical Adherence and Missing Appointments, and (**D**) Video 4: A Couple with Obesity

The scripts were submitted to the team hired to develop the animation in the production phase. Then, cartoon characters and backgrounds were designed, and images, text, voice acting, and music to enhance engagement and attraction to patients were produced. Finally, 4 animated videos that combined visual, audio and used of subtitles were developed, which included the 1st video, Diabetes and Eating Habits of a Working Woman (4.14 min), the 2nd video, Hypertension, and Risk Factors (2.18 min), the 3rd video, Medical Adherence and Missing Appointments (3.03 min), and the 4th video, A Couple with Obesity (3.16 min). Each video described different real situations in daily life, followed by seeing the physician for treatment plans, and recommendations for appropriate self-care when sick. Subtitles were included in the video to increase understanding. Then, a summary of recommendations was provided as the on-screened text at the end of each video. (Fig. 2)

In the post-production phase, the four videos of 2D short animations were validated by the physicians for correct information on signs and symptoms of the diseases and their treatment and behavior change recommendation, and by media experts for images and motion graphics. After completing the videos, experts were asked to review and revise them until the research team was satisfied.

### Study sample and recruitment

Adults with diabetes and/or hypertension from two Health Promoting Hospitals in 2 provinces were recruited by the researchers to attend the focus groups. Inclusion criteria included those subjects being adults 35–70 years old, having smartphones, being diagnosed with diabetes or hypertension, and being able to communicate in Thai.

### Data collection

Eligible participants were invited to participate in the study. They were informed of the study’s purpose, explained in detail, and addressed any questions they had by the researchers. Importantly, all participants were informed that participation was voluntary and those who were interested were asked to sign the written IRB approval informed consent. All participants received approximately US$ 3 for their participation in the study.

Participants were asked to complete a sociodemographic questionnaire including sociodemographic characteristics, illness history (duration, admission, complication, and so forth), and risk behaviors (smoking and alcohol drinking). Four 2D short animated videos were shown one at a time by the researchers with using the researchers’ smartphones, followed by a discussion guided by semi-structured interviews and probing questions. All interviews were conducted at the primary care clinics, where they received routine care, from the researchers who have been trained for qualitative data collection. The focus-group sessions were composed of 3–4 people each time, lasting from 25 to 35 min, and were transcribed verbatim. Information was digitally recorded on smartphones. According to the study plan, ten participants were proposed to be recruited per setting in each province due to the healthcare service and community context concerns. After completion of the interview with ten participants in the first setting, the information findings were discussed among the researchers to identify initial keywords or phrases before repeating the interview process in the next primary care clinics. Finally, the interviews were ended after 20 participants from two primary care clinics in two suburban provinces because of data saturation, meaning no more new information could be reached.

#### Interview guide

A semi-structured interview was developed for the study by the researchers. It included the following four main questions: *What messages do the videos highlight for you? Would you prefer changes to the videos*,* including content*,* and for what reasons? Do you feel these videos motivate you or might motivate others like you to change behaviors? If you have an opportunity to develop any animated video*,* what do you have in your content*,* length of the video*,* and picture?* Also, probing questions were provided to get further information.

### Data analysis

Thematic analysis was used to identify patterns of meaning across an entire qualitative data in the study [23, 24]. Emerging themes and subthemes were developed from the information deductively using coding. Initially, the research team identified and labeled the participants’ descriptions with codes in each video by two researchers who have experience in qualitative research. Then, all codes were grouped into subthemes and themes in an ongoing inductive process until consensus was established. No software programs were used to assist with this thematic analysis.

## Results

Twenty persons, one man and the nineteenth woman, who met the criteria agreed to participate in this study. As shown in Table 1, the mean age was 58.4 years (SD 6.7), ranging from 41 to 68 years. The majority of them were married (65%), finished primary school level (65%), and unemployed (65%). Among participants, there were 35% diabetes, 35% hypertensive, and 30% diabetes and hypertensive patients. The average years of diabetes was 8.5 years (SD 5.7, ranged 1–20 years), and for hypertension was 8.1 years (SD 5.4, ranged 1–20 years).

**Table 1:** Sociodemographic characteristics (*N* = 20)

Characteristics	
Age (years), mean (S.D); min - max	58.4(6.7); 41–68
Gender (n, %)	
Male	1(5)
Female	19(95)
Education level (n, %)	
None	1(5)
Primary school	13(65)
High school	6(30)
Marital status (n, %)	
Not married	1(5)
Couple	13(65)
Separate/widow	6(30)
Occupation (n, %)	
Unemployed or work at home	13(65)
Employed	7(35)
Illness history Diabetes (n, %); years (mean (S.D); min – max)	35%; 8.5(5.7); 1–20
Hypertension (n, %); years **(**mean (S.D); min – max)	35%; 8.1(5.4); 1–20.
Diabetes and hypertension (n, %)	30%

The findings from the focus-group interview analyses for each video were presented through the three themes—presentation, impacts, and suggestions—with subthemes. (Table 2)

**Table 2:** Themes, subthemes, and quotations in four videos

Themes	Subthemes	Supporting quotations			
		Video 1	Video 2	Video 3	Video 4
Presentation	Images and Graphic motion	There’s good visual motion that’s simple to follow. (Female, 65)	Older adults enjoy images such as this. (Female, 56)	There are not too many letters, yet the image and sound are both well visible. (Female, 61)	Cartoons offer excellent action and visuals. The sound is audible. (Female, 50)
	Length	It is the perfect length for the video 1. It seems like this video would be harder to follow if certain parts were removed, even though the way it was presented works well. (Female, 49)	People will become impatient if it is too long, therefore this is a fair length. (Female, 67)	This kind of content’s length is good. Viewers will become impatient if we go on for too long. (Female, 62)	The length is acceptable; it doesn’t require much time to watch and is easy to understand. (Female, 58)
	Language/sound	It’s easy to watch and understand	The speech was clear, easy to understand, and not too long. Beautiful character. (Female, 58)	The videos have a clear voice and a slow pace for hearing. (Female, 62)	The animation uses language that is easy to understand. Don’t use too many medical terms, which makes it easy for us to understand. (Female, 58)
	Content	In the first video, it talks about what we should eat and what we shouldn’t eat, and exercise. (Male, 65)	In the second video, illustrations of teenagers fit people these days, and I can understand the consequences of alcohol drinking. (Female, 62)	In the third video, I got the idea that we shouldn’t miss the medicine. The video was right. We shouldn’t miss the medicine at all. (Female, 63)	The fourth video suggests us to reduce starch and eat vegetables, fruits, and things like that… It teaches how to control diet. (Female, 58)
		Our behavior is similar to that in the video 1. It is our everyday existence, where everyone is mostly dependent on sugar-sweetened foods and drinks, whether it be bubbling tea, fruit, or anything else. (Female, 49)	It is a good video; that is, the friends warn people not to drink or smoke. (Female, 58)	The mother accepted the snacks her child had purchased her and consumed them. (Female, 49)	According to the video’s content, consuming starchy and fatty foods makes you gain weight. Reduced eating, less cravings, and weight loss are all necessary. (Female, 41)
Impacts	Recalling information	The cartoon shows the typical day-to-day activities of people. This is how we all live…There’s no need for improvement because it’s easily understood. (Female, 67)	After watching cartoons, I feel that I have more knowledge about eating and living. It makes me want to adjust such as eating habits or not drinking. (Male, 65)	Taking medication as prescribed, keeping doctor’s appointments, and continuing to take the medication are all important. (Female, 41)	It is said that eating anything that can increase your body weight is not a good idea…It is recommended to eat vegetables and things like that. (Female, 56)
	Enhancing engagement	It reflects on me a lot. Because every season that durian comes out, we like to eat them and my blood sugar always rises. I was constantly warned about this issue by the doctor. (Female, 63)	The video reminds me of my grandson-in-law. We found that his blood pressure was very high, and his sister said that he drinks beer every day. (Female, 62)	The content is just like what the doctor recommended. (Female, 63)	It makes me think of myself when I look at it. I’d like to eat this and that sometimes. (Female, 41)
		I am encouraged to be more curious by cartoons. If it were a live-action character, it would not come across a lot, and it would seem normal without any motivation. (Female, 67)	I think about people I’ve talked to in the past after seeing it. He stated he was afraid to visit the doctor because he believed the physician would forbid him from drinking alcohol. (Female, 41)	My thoughts are reflected in the text. I always take food that my child offers me. (Female, 65)	The physician describes information without showing images. However, when I view it this way, the image becomes visible. (Female, 56)
	Motivating health awareness and behavior change	When I see this animation about diabetes, I will be able to improve myself, even though I have not been diagnosed with it. I have hypertension. (Female, 63)	After watching cartoons, I feel that I have more knowledge about eating and living. It makes me want to adjust, such as eating habits or not drinking. (Male, 65)	The content of having desserts a lot is exactly me. After looking at it, I realized that I should reduce it somewhat so I would get better. (Female, 63)	I realized I needed to change after watching the videos. Give up eating fried, oily, or similar foods…It’s quite helpful. I believe that if individuals watched these videos, their behavior might change since they would undoubtedly become more cautious about becoming sick.” (Female, 58)
Suggestions	Enlarging font size	The last slide couldn’t be read in time. The font is tiny and I have a bad sight. (Female, 62)	If you’re over 60, you may need to enlarge the lettering slightly when viewing it on a smartphone, but otherwise, the letters remain legible. (Female, 49)	The letters are tiny and unreadable. (Female, 63)	I hardly see text if I read it myself.
	Changing to spoken text summaries	About the last summary content, it can’t be read in time. it should be spoken so I’ll hear it immediately. (Female, 63)	It should be spoken rather than written. Older people like us have to read it over and over again with eyeglasses on. (Female, 61)	It was difficult to read the video’s conclusion at the end. Since we are elderly, it would be preferable if you spoke loudly. (Female, 56)	The text is readable for me. Speaking loudly is preferable nevertheless, as it won’t be simple to comprehend. (Male, 65)
	Favorite	Watching cartoons is easy to remember. Animation has pictures, that are easy to understand and speak clearly. (Female, 68)	What I especially like is the advice to abstain from drinking. (Female, 49)	When the doctor teaches me, it is beneficial. To find out, though, I must visit a doctor. I can still watch cartoons at home so I can still learn from them. Cartoons are something I can watch on my phone. (Female, 61)	Cartoons provide us with more comparisons to look at than live actors play because they offer images of obese people. (Female, 56)

### Presentation

This theme reported participants’ positive responses in images, graphic motion, length, and language/sound. Overall, most participants indicated that the content in videos is understandable because the language does not use medical terms. Also, the speech of the video dialogue is clear and slow, which makes it easy to follow the content. Importantly, the majority of participants can understand video content and its intended purpose with accuracy.

Image and graphic motion.

There’s good visual motion that’s simple to follow. (female, 65)

Cartoons offer excellent action and visuals. The sound is audible. (female, 50)

Language/sound.

*“The animation uses language that is easy to understand. Don’t use too many medical terms*,* which makes it easy for us to understand.” (Female*,* 58)*.

Content

*“In the third video*,* I got the idea that we shouldn’t miss the medicine. The video was right. We shouldn’t miss the medicine at all.” (Female*,* 63)*.

Noticeably, although the video on Diabetes and Eating Habits of a Working Woman was 4.14 min long, participants indicated that it seemed harder to follow if some of the content was removed. None of them have suggested any changes in the length of the video presentation: *“It is the perfect length for the video 1. It seems like this video would be harder to follow if certain parts were removed*,* even though the way it was presented works well.” (Female*,* 49)*.

### Impacts

Overall, participants indicated the significant impacts of animated videos; that is, they perceived that the animated videos helped them remember the advice on behaviors they had been given when visiting the clinics: *“The content is just like what the doctor recommended.” (Female*,* 63)*. Specifically, in the first video, participants reported that animations were more engaging in video situations than live actors: *“I am encouraged to be more curious by cartoons. If it were a live-action character*,* it would not come across a lot*,* and it would seem normal without any motivation.” (Female*,* 67)*.

In the 2nd and 3rd videos, the cartoon features and narratives made the videos more engaging in the situations that they are familiar and brought up memories of past experiences. Following their viewing of all videos, they also reported intentionally positive changes in their health awareness and behavior:

*“After watching cartoons*,* I feel that I have more knowledge about eating and living. It makes me want to adjust*,* such as eating habits or not drinking.” (Male*,* 65)*.

*“I realized I needed to change after watching the videos. Give up eating fried*,* oily*,* or similar foods…It’s quite helpful. I believe that if individuals watched these videos*,* their behavior might change since they would undoubtedly become more cautious about becoming sick.” (Female*,* 58)*.

### Suggestions

Participants emphasized a few recommendations for improvement. Some participants thought that if the videos were meant to be seen on a smartphone, the font size ought to be increased. The majority of participants reported having trouble seeing the information text: *“If you’re over 60*,* you may need to enlarge the lettering slightly when viewing it on a smartphone*,* but otherwise*,* the letters remain legible.” (Female*,* 49)*.

Similarities in all videos have been observed. It was suggested that the videos would be more beneficial if the summary of suggestions at the conclusion of the videos included spoken text: *“About the last summary content*,* it can’t be read in time. it should be spoken so I’ll hear it immediately.” (Female*,* 63)*.

There are some different interesting findings of each video to support that animated videos are potential strategies to enhance health literacy among adults with chronic illness. First, graphic displays can facilitate participants learning in performing complex behavior changes and portray abstract concept to visual such as obese people: *“Cartoons provide us with more comparisons to look at than live actors play because they offer images of obese people.” (Female*,* 56)*.

Secondly, participants can access to animations that can be viewed anywhere at any time using a smartphone if they need health-related information:*“When the doctor teaches me*,* it is beneficial. To find out*,* though*,* I must visit a doctor. I can still watch cartoons at home so I can still learn from them. Cartoons are something I can watch on my phone.” (Female*,* 61)*.

## Discussion

Adults can be self-directed learners when their motivation is enhanced through reasons for engagement and overcoming their barriers [25, 26]. Animated videos are promising in grasping people’s attention with images and sound and making complex healthcare practices more understandable. The study explored the perceptions of adults with diabetes and hypertension toward four animated videos viewed on smartphones to enhance literacy about chronic diseases.

Findings demonstrated participants’ positive responses regarding the suitability of images that reflect their daily living, the good flow of information with the smooth graphic motion, and its shortness. In the study, each video content was classified as displaying improper practice and its consequence, physician recommendations, and a summary of recommended behavior, and the video length ranged from 2.18 to 4.14 min. Video animations were consistent with previous findings that compelling animated videos should be based on real-life situations and learner-centered interests and be short with manageable detail [11, 27, 28].

Most participants perceived that although healthcare professionals have provided most content while receiving care at the primary care clinics, the animated videos provide a more profound understanding of knowledge/information. Similar results were found by Stebner et al., indicating that people who learned from animations could better comprehend the content than those who learned from static pictures [29]. This educational advantage is consistent with another study that found that spoken animation enhanced patients with low literacy in recalling health information [27, 30]. This finding supported the Cognitive Theory of Multimedia Learning that visualization combined with text overcomes low literacy and facilitates learning [14]. 

It becomes clear that they could identify several risk factors and improper behaviors shown in the videos for getting chronic diseases, such as eating habits and alcohol drinking. Moreover, most participants indicated that this content motivated them to think about planning to change or recommend such behaviors to others. This motivation might be because the videos used simple language without medical terms to explain the kind of improper behaviors, explain signs and symptoms of the diseases, and recommend the behavior changes they can do every day. This finding demonstrated the importance of animated videos as a learning tool to convey complex messages about chronic diseases and healthy habits [11, 27, 31]. 

Participants perceived that animation helps them become more cautious about getting sick, even though some video content was irrelevant to their health conditions. In the study, four differences in cartoon characters (age and gender) and several patients’ diseases were created and shown to participants. This mix of characters was inconsistent with the findings from a meta-analysis of 21 published animated videos in clinical trials, which concluded that animations should be developed for a specific patient population for better learning [11]. Compared to live action, which is linked with reality and encourages the less interactive study of the information, animations more easily link the audience to a world of fantasy, which leads to their engagement and more curiosity towards the information [32]. 

The targeted population’s preferences should be considered when developing animated videos to enhance the audiences’ openness to the health message. In the study, participants suggested improving the font size for subtitles to a bigger size. This suggestion might be because the videos were viewed on smartphones, which have a limitation on the display screens. In addition, they proposed that the researchers consider spoken text instead of on-screen text for recommendations at the end. Having spoken text is consistent with findings in a study by Schüler, indicating that spoken text is highly recommended to complement information and allow better learning compared to written text because it reduces the cognitive demands and time on the learning material [33]. Therefore, the videos have been revised based on the participants’ feedback. However, subtitles and written messages are still displayed on videos because subtitles and written messages benefit people with difficulty hearing [34]. 

### Future research

Learning more about what videos the patients decide to watch on their own and any motivations behinds their choices might be beneficial. Although the positive feedback was provided for all videos, it might be they were new in the primary care clinic in terms of delivery health message, which might be attractive at first glance. In addition, all videos were provided by the researchers with the purpose of exploring their understanding and finding weak points of the videos, not requiring for their selection. Therefore, further study should be conducted to investigate an effectiveness of this strategy to enhance behavior changes compared to the traditional education in adults with chronic diseases who receive care in the primary care clinics.

### Strengths and limitations

The primary strength of the study is that the animated videos were developed and validated by interdisciplinary experts, including medicine, nurses, health education, and technology. Another strength is using real-life situations from nursing field practice and clinical practice guidelines for writing scripts. Using examples allows participants to increase uptake of and engagement with signs and symptoms of the diseases and recommendations for self-care.

This study has several limitations for generalization. First, the majority of participants were elderly females. Although the information from the interview captured ideas from the main population with chronic diseases and low literacy in our communities, additional suggestions from the younger age group and males could be valuable and confirm these findings. Second, while the video animations were validated in terms of the depth of the content and the presentations from experts, further research is needed to assess the effects of animations on health literacy and self-care and practically applicable access via smartphones.

## Conclusion

Animated videos for Literacy Against Chronic Diseases for adults with diabetes and/or hypertension are developed. Positive responses resulted from the animated video presentations and their impacts. They were rated as having suitable images and motion graphics, being short enough, with simple language, clear sound, understanding content, recalling information, enhancing engagement, and motivating health awareness and behavior change. Feedback from adults with diabetes and hypertension for final improvement included font-size increments and spoken-text summaries. The future study should aim to investigate the effectiveness of animated videos to increase health literacy and behavior changes among adults with chronic diseases.

## Data Availability

The datasets used and analyzed during the current study are available from the corresponding author on reasonable request. Status of data and materials: Data are available on request due to privacy/ethical restrictions Statement: The data that support the findings of this study are available on request from the corresponding author, [Pichayapinyo, Panan: panan.pic@mahidol.ac.th]. The data are not publicly available due to [restrictions e.g. their containing information that could compromise the privacy of research participants].Status of data and materials: Data and animated videos are generated and available upon request Statement: Derived data and animated videos supporting the findings of this study are available from the corresponding author [Pichayapinyo, Panan: panan.pic@mahidol.ac.th] on request.Status of data and materials: Data available within the article or its supplementary materials Statement: The authors confirm that the data supporting the findings of this study are available within the article.Status of data and materials: Data available on request from the authors Statement: The data that support the findings of this study are available from the corresponding author, [Pichayapinyo, Panan: panan.pic@mahidol.ac.th], upon reasonable request.
